# Tissue stiffness in BPH patients from magnetic resonance elastography

**DOI:** 10.1186/s43055-021-00679-8

**Published:** 2021-12-13

**Authors:** Chalida Aphinives, Watcharaphon Kiatsayompoo, Kulyada Eurboonyanun, Prin Twinprai, Saranya Jaruchainiwat

**Affiliations:** Department of Radiology, Faculty of Medicine, Khon Kaen University, 123 Group 16, Mittraparp Rd, Muang, 40002 Khon Kaen Province Thailand

**Keywords:** BPH, MRE, Tissue stiffness, Shear wave, Noninvasive

## Abstract

**Background:**

BPH is commonly found in older men which can lead to lower urinary tract symptoms. Magnetic resonance elastography (MRE) is an innovative, noninvasive imaging technique used to evaluate tissue stiffness. There has not been any study, however, that assessed the tissue stiffness in patients with BPH. A prospective descriptive study was performed to demonstrated MRI and MRE techniques of the prostate gland in ten patients with BPH to assess tissue stiffness, features of BPH on MRI and components of BPH in the area of increased stiffness.

**Results:**

MRI and MRE examinations in all patients were successful without any complications. The mean tissue stiffness of the whole prostate gland was 4.40 ± 0.71 kPa with good reproducibility (ICC 0.82). Stromal components and mixed glandular-stromal components tended to be associated with the areas of increased stiffness on stiffness images, 50.6% for stromal components and 37.9% for mixed glandular-stromal components. Some MRI findings were seen on the patients with high mean stiffness values such as prostatic calcification, type-5 BPH pattern and large prostate volumes.

**Conclusions:**

Prostate MRE is a useful noninvasive reproducible diagnostic tool for evaluating prostate tissue stiffness by both qualitative and quantitative assessments. The mean prostate tissue stiffness from MRE in patients with BPH in this study was 4.40 ± 0.71 kPa. Some MRI features might be associated with increased tissue stiffness.

*Trial registration*: PID 229. Registered 4 October 2019. http://md.redcap.kku.ac.th

## Introduction

Benign prostatic hyperplasia (BPH) is a condition commonly found in older men. It can be found in 50–60% of men by the age of 60 [1] and can lead to lower urinary tract symptoms (LUTS), including voiding and storage symptoms. About 90% of men aged between 45 and 80 have at least one LUTS [2].

BPH can be divided into two main components; glandular and stromal enlargements [3]. Glandular enlargement mainly leads to an increase in the prostate gland’s size, resulting in obstructive symptoms. In contrast, stromal enlargement leads to increased resistance of prostatic parenchyma, which is known as the dynamic effect [4]. BPH initially occurs in the periurethral gland (PUGs) and then continues to the transition zone (TZ), which is the main site of BPH [5]. Although BPH mainly develops in TZ and PUGs, it can also affect the peripheral zone [6]. Randall et al. classified patterns of BPH according to their MRI appearances into eight types [7].

The Prostate Imaging Reporting and Data System (PI-RADS) has recommended that the measurement of the maximum AP and longitudinal diameters should be made on the mid-sagittal T2W image, and the measurement of the maximum transverse diameter should be made on the axial T2W image [8]. The size of the prostate gland can be used to determine the treatment options.

Magnetic resonance imaging (MRI) is frequently used for the detection and risk stratification of clinically significant prostate cancer (csPCa) because of its higher resolution than other modalities [9]. BPH patients would be requested for MRI assessment when they have an abnormally high prostate-specific antigen (PSA) level or clinical suspicion of prostate cancer [10].

Magnetic resonance elastography (MRE) is an innovative, noninvasive imaging technique. It can provide information about the biomechanical properties of soft tissue and quantitative assessment based on different stiffnesses between healthy and diseased tissues. The most widely used application of the MRE is detecting and staging liver fibrosis, which is currently performed in more than 100 centers worldwide [11].

Many recent studies have assessed the utility of MRE in other organs such as the brain, muscle, and breast [12–15]. Only a few studies have assessed the utility of MRE in the prostate gland.

Kemper et al. assessed the feasibility of MRE in the prostate gland of seven healthy volunteers, using an external driver attached to the pubic bone on a 1.5 T scanner at a vibration frequency of 85 Hz, and found that the prostate gland had significantly higher stiffness than the adjacent fat tissue [16]. After that Sahebjavaher et al. studied MRE using 3.0 T scanner and transperineal electromechanical transducer at a frequency of 70 Hz in six healthy volunteers and found a higher shear stiffness of the central and transition zones than the peripheral zone [17]. According to the study of Dittmann et al. to assess the elasticity of prostate gland in 12 healthy volunteers using MRE and three externally placed pressurized-air drivers at vibration frequencies of 60, 70, and 80 Hz, there were no significant differences of values of shear wave speed between the peripheral zone and the central gland. They also assessed MRE in patients with prostate cancer and found no significant differences in shear wave speed from those of healthy volunteers. In two prostate cancer patients, however, there were areas of obviously increased stiffness that were distinct from the remaining prostate tissue [17].

There has not been any study that assessed the tissue stiffness in patients with benign prostatic hyperplasia. Therefore, a prospective descriptive study to demonstrate the technical feasibility of prostate MRE using an external air driver and assess the stiffness of prostate tissue along with MRI features of the enlarged prostate gland in patients with BPH was conducted.

## Materials and methods

### Ethical consideration

This study was a prospective descriptive study from November 2019 to October 2020. The study protocol was approved by the Ethics Committee for Human Research.

### Study population

The inclusion criteria were (1) clinical or MRI features of BPH, (2) PIRADS (v. 2.1) score 1 or 2.

The exclusion criteria were (1) prostate cancer (2) MRI artifacts that would affect an imaging interpretation.

Ten patients were enrolled in the study. Written informed consent was obtained from all the subjects.

### Hardware and data acquisition

All examinations were performed in a 3.0-Tesla MRI scanner (Achieva dStream, software version 5.6.1.0, Philips Healthcare) using a phased-array surface coil. To perform MRE and generate mechanical waves, a pneumatic system that incorporated an active driver (placed outside the scan room) for producing continuous acoustic wave motion was used. A passive driver was placed against the body surface superficial to the pubic symphysis to induce shear waves traversing the prostate gland. An air-filled plastic tube was used for transmitting the pneumatic excitations from the active to the passive driver. A small soft pad was also placed beneath the passive driver to decrease the patient’s vibrating sensation. The patient placed in the supine position, then a small soft pad and a passive driver are placed over the pubic symphysis. The passive driver and the pelvis were wrapped together by a kidney belt to ensure direct contact of the driver with the body surface. Finally, an anterior surface coil was set up before performing an MRI examination. A repetition frequency of the drivers at 60 Hz was used for the MRE study.

Prostate MRI was performed using the routine prostate protocol as demonstrated in Table 1. A gadolinium-based contrast was administered if there were no contraindications.

**Table 1:** MRI protocol

Sequence	TR	TE	FA	Thickness/gap	FOV	Matrix	NEX
Coronal T2W GRE	2.8	1.41	60	6/-3	360*360 (Entire pelvis)	232*228	1
Sagittal T2W	3360	100		3/0	170*170 (Entire prostate gland)	284*233	1
Coronal T2W	2500	100		3/0	180*180 (Entire prostate gland)	256*239	1
Axial T2W	3561	100		3/0	160*160 (Entire prostate gland)	268*217	2
Axial T2FS	4000	75		3/0	160*160 (Entire prostate gland)	268*235	2
Axial T1W	587	10		3/0	160*160 (Entire prostate gland)	268*225	1
Axial DWI and ADC map using b-value of 0, 800, 1000 and 1500 s/mm^2^	4472	68		3/0	180*180 (Entire prostate gland)	92*101	4
Axial T1W	654	10		6/1	360*360 (Entire pelvis)	360*239	1
Axial T2W	2651	110		6/1	360*360 (Entire pelvis)	300*300	1
DCE in axial plane with a temporal resolution of 8 s	4.9	1.95/3.3	10	3	180*200 (Entire prostate gland)	144*160	1
T1FS post Gd on axial plane	572	8		3/0	360*320 (Entire pelvis)	276*207	1
T1FS post Gd on coronal plane	600	10		3/0	180*180 (Entire prostate gland)	256*198	1
T1FS post Gd on sagittal plane	772	16		3/0	170*170 (Entire prostate gland)	256*177	2

To image the shear waves and measure the propagation of the mechanical waves inside the tissue from the MRE, a 2D Phase Contrast Fast Field Echo (FFE) with motion-encoding gradients and a 15-s breath-hold per slice was applied. Depending upon the prostate gland size, two to five axial slices through the whole prostate gland were generally obtained to generate MRE images. Before imaging the shear wave, axial T2W images were acquired to position the prostate gland and to be used as an anatomical reference. Each wave image slice was acquired at eight evenly spaced time points. The MRE protocol is shown in Table 2. The MRE examination was repeated once without repositioning the patient or the actuators to assess reproducibility.

**Table 2:** MRE protocol

Field of view	360 mm × 318 mm
Matrix	240 × 82
ACQ voxel size	1.5 × 4.5 mm
Slice thickness	10 mm
No. of slices	2–5 slices (depending on the size of prostate gland)
Flip angle	30°
TR	50
TE	20
Breath-hold	15 s

### Data post-processing

**Fig. 1:**
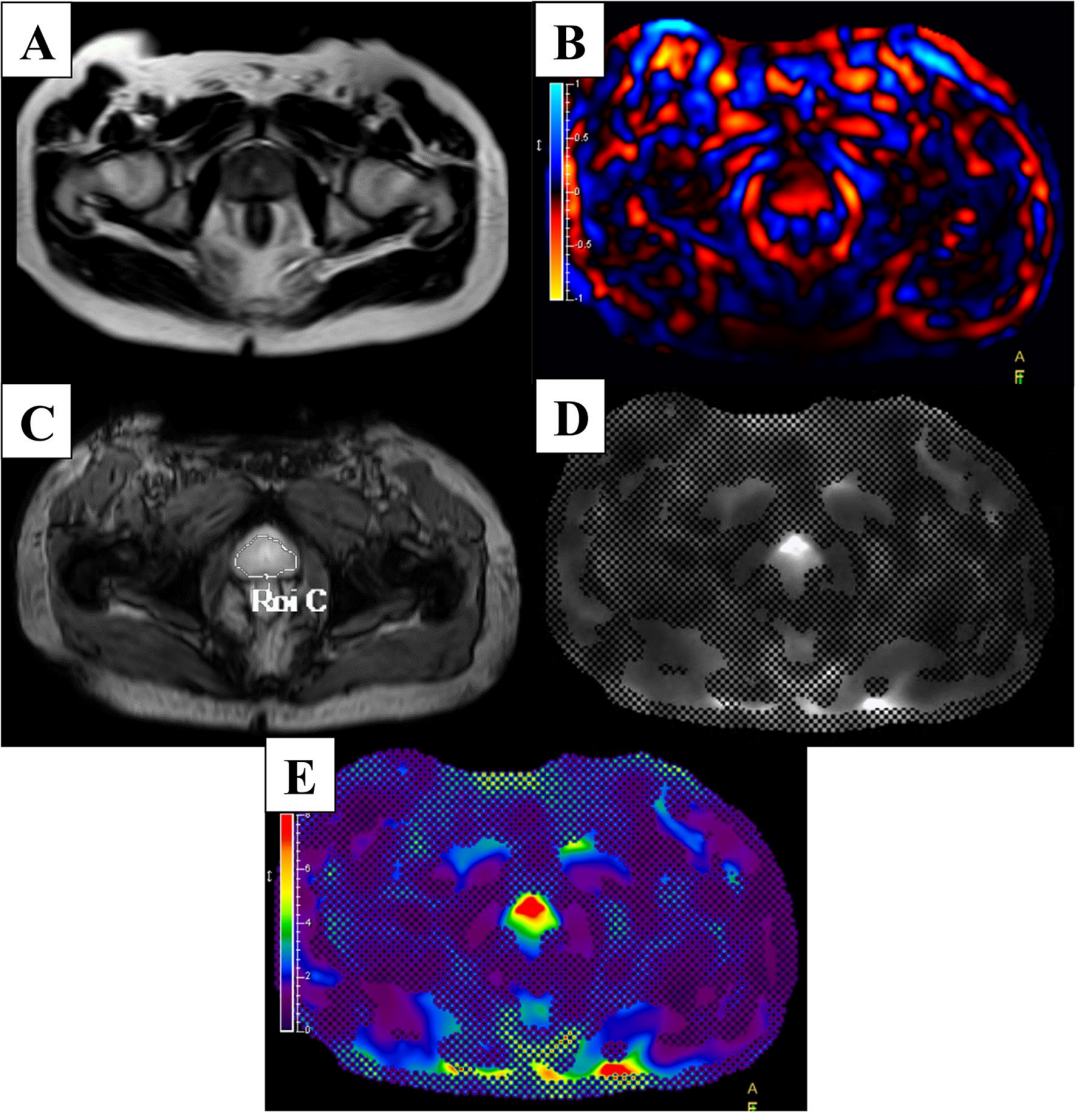
Demonstrating MRE images in one same slice. **A** T2W image. **B** Wave image. **C** FFE image. **D** Modulus image. **E** Stiffness image

**Fig. 2:**
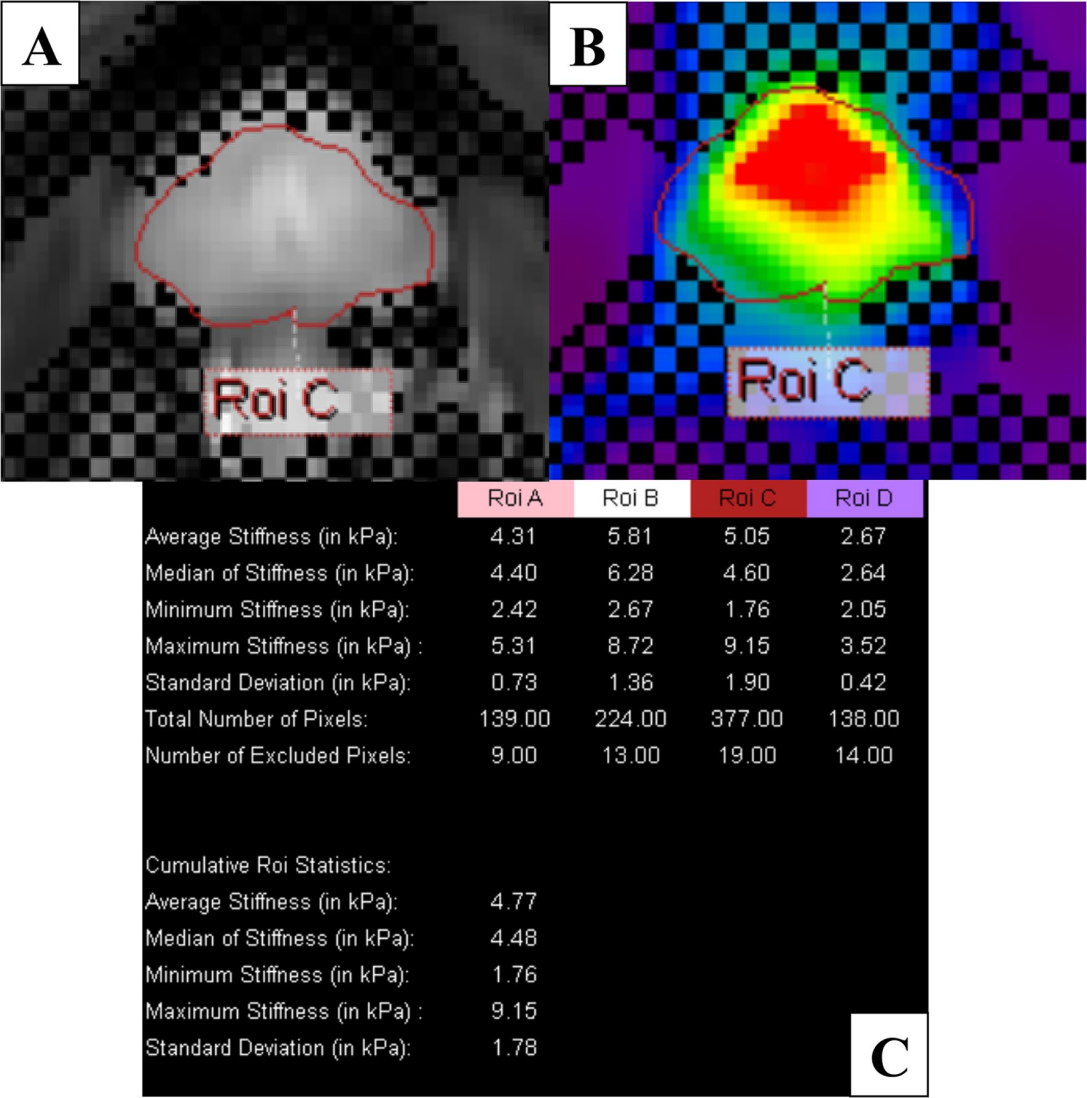
Demonstrating the measurement method and documented results in post-processing software. **A **The ROI was drawn manually covering the entire prostate gland on the axial image. **B** ROI was also shown on the stiffness image. **C** Displayed stiffness values in each slice in average, median, minimum, maximum, and SD of stiffness with aggregate stiffness values

Post-processing of the images displaying wave images, FFE/Modulus images and stiffness images was performed using MREView software (software version 5.6.1.0, Philips Healthcare) (Fig. 1). The regions of interest (ROIs) for the whole prostate were drawn manually on axial FFE/Modulus images by an MRI technologist to measure tissue stiffness (Fig. 2). Each pixel on an MRE image was processed by the software and converted into stiffness values. All pixels in the ROI were calculated to achieve overall stiffness values. The stiffness measurements in units of kilopascals (kPa) were displayed as average stiffness, median stiffness, minimum and maximum stiffnesses, and standard deviation (SD). The stiffness images were displayed in color where red color represented high stiffness and a dark purple color represented no stiffness.

### Imaging interpretation

Two abdominal radiologists with 9 and 23 years of experience reviewed MRI and MRE images by consensus. Both radiologists were blinded to stiffness values.

The axial thin-slice T2W images through the whole prostate gland were used to determine the components of BPH. The proportion of BPH was classified into three categories: (1) glandular predominate (glandular component > 50%), (2) stromal predominate (stromal component > 50%), and (3) equal glandular and stromal component. The BPH pattern was classified according to Randall’s classification [10]. The dimensions and volumes of the prostate gland were measured and recorded in centimeters (cm) and milliliter (ml). Other findings seen on MR images such as hemorrhage or utricle cyst were also recorded.

For the MRE assessment, the number of acquired slices, mean stiffness with SD, minimum and maximum stiffness (kPa) were recorded. The stiffness maps and T2W images were simultaneously interpreted to evaluate the components of BPH in the area of increased stiffness, which was defined by red or orange colors in the stiffness map.

### Statistical analysis

Continuous data were demonstrated by their means and SD, or median and interquartile range. Categorical data were demonstrated by number and proportion. Comparisons of numerical data between groups were analyzed by using a nonparametric test. The intraclass correlation coefficients (ICC) between the measurements of two MRE examinations to evaluate reproducibility were done. The level of significance was set at *p* < 0.05.

## Results

Eight patients had PI-RADS scores of 2 and two patients had PI-RADS scores of 2/3 due to disagreement between two reviewers. None of them had a PI-RADS score of 1. Baseline characteristics are displayed in Table 3. The average PSA level was 11.85 ng/ml (SD 8.43, range 2.11–26.70 ng/ml). The average volume of a prostate gland was 90.1 ml (SD 61.82, range 51.21–239.73 ml). There was a significant correlation between the PSA level and the volume of the prostate gland with a moderate positive correlation (*r* = 0.590; *p* = 0.021). The PSA level had no significant correlation with the prostate height (*p* = 0.383) or proportion of BPH (*p* = 0.237). Two patients underwent biparameter MRI (bpMRI) and eight patients underwent multiparameter MRI (mpMRI) before MRE examination. All subjects tolerated the mechanical vibration and scan protocol well. None had any complications from the MRI/MRE examination. The MRE examination time in each patient was less than 20 min.

**Table 3:** Patient characteristics

	*n*	Average	SD	Min	Max	Median
Age (year)	10	70.6	10.4	57.0	91.0	70.5
BMI (kg/m^2^)	10	24.9	3.7	19.1	29.8	24.6
PSA level (ng/ml)	10	11.85	8.43	2.11	26.70	9.74
Prostate width (cm)	10	5.72	0.73	4.84	7.11	5.60
Prostate height (cm)	10	6.11	1.97	4.53	10.99	5.34
Prostate AP (cm)	10	4.53	0.78	3.71	5.90	4.23
Prostate volume (ml)	10	90.1	61.8	51.2	239.7	65.7
PSAD (ng/ml^2^)	10	0.14	0.08	0.03	0.33	0.16

From T2W images, there were four patients with a predominant glandular component, three patients with predominant stromal component, and three patients with equal glandular-stromal component. Nine patients had bilateral TZ and a retrourethral enlargement pattern (type 3) and only one patient had a pedunculated, bilateral TZ and retrourethral enlargement pattern (type 5) (Table 4).

**Table 4:** MRI features of all patients in the study

No	Protocol	Proportion of BPH	Pattern of BPH	PIRADS	Prostate volume (ml)	PSA (ng/ml) PSAD (ng/ml^2^)	Other findings
1	mpMRI	Glandular predominant	Bilateral TZ and retrourethral enlargement (type 3)	2	63.4	10.30 0.16	Chronic prostatitis and scar at left peripheral zone of mid gland
2	mpMRI	Glandular predominant	Bilateral TZ and retrourethral enlargement (type 3)	2/3	53.1	9.17 0.17	Hemorrhage at right transition zone of mid gland (1.5 × 1.2 cm)
3	mpMRI	Glandular predominant	Bilateral TZ and retrourethral enlargement (type 3)	2	68.1	2.11 0.03	Hemorrhage at left transition zone of mid gland (1.2 × 0.3 cm)
4	mpMRI	Stromal predominant	Bilateral TZ and retrourethral enlargement (type 3)	2	58.7	7.73 0.13	
5	mpMRI	Equal	Pedunculated with bilateral TZ and retrourethral enlargement (type 5)	2/3	239.7	11.04 0.04	A 0.8-cm prostatic utricle cyst with internal hemorrhage/high proteinaceous content Several calcification foci A small area of hemorrhage at right TZ of mid gland
6	mpMRI	Equal	Bilateral TZ and retrourethral enlargement (type 3)	2	72.5	13.10 0.18	Minimal ascites in pelvic cavity
7	bpMRI	Stromal predominant	Bilateral TZ and retrourethral enlargement (type 3)	2	161.2	26.70 0.17	Minimal ascites in pelvic cavity
8	bpMRI	Equal	Bilateral TZ and retrourethral enlargement (type 3)	2	51.3	4.08 0.08	A prostatic utricle cyst 0.5 cm
9	mpMRI	Glandular predominant	Bilateral TZ and retrourethral enlargement (type 3)	2	51.2	7.66 0.15	A few small high SI foci on T1W; probably calcification, high-proteinaceous content or hemorrhage
10	mpMRI	Stromal predominant	Bilateral TZ and retrourethral enlargement (type 3)	2	81.5	26.60 0.33	Minimal ascites in pelvic cavity

**Fig. 3:**
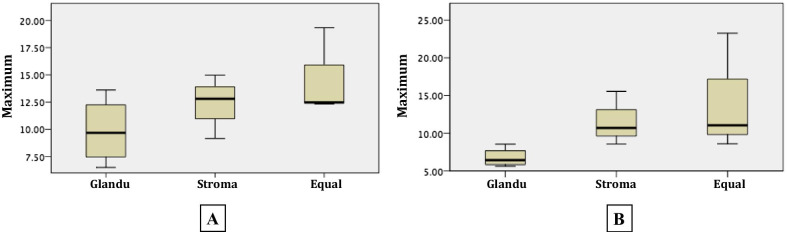
Demonstrating correlation of the maximum stiffness (kPa) between each predominated proportion of BPH in **A** first MRE **B** second MRE

The mean stiffness of the whole prostate gland was 4.40 kPa (SD 0.71, min 3.35, max 5.96 kPa) (Table 5). Patients with glandular predomination tended to have lower maximal stiffness than stromal predomination or equal glandular-stromal components (*p* = 0.317 in first MRE and 0.035 in second MRE) (Fig. 3). There were no significant relationships between the average, median and minimal stiffness and different types of BPH. It was found that only one patient (No#4) had different acquired slices, four slices in first MRE and three slices in second MRE. There was an excellent correlation between the number of slices in first and second MREs (*r* = 0.93; *p* = 0.001). The number of acquired slices correlated with the height of the prostate gland. The group of 1–3 slices-acquired had an average prostate height of 4.90 ± 0.41 cm and the group of more than three slices-acquired had an average prostate height of 6.42 ± 3.46 cm (*p* = 0.08 for the first MRE and *p* = 0.01 for the second MRE). There were 87 areas of increased stiffness on stiffness images (43 from first MRE and 44 from second MRE). Forty-four areas (50.6%, 22 from first MRE and 22 from second MRE) corresponded to a stromal component on T2W images. Thirty-three areas (37.9%, 16 from first MRE and 17 from second MRE) corresponded to mixed glandular and stromal components. There was one area that was seen only on the second MRE, whereas, other areas were the same areas as seen on the first MRE. There were only four areas (4.6%, two from first MRE, and two from second MRE) that corresponded to the glandular component. Four areas in patient No#2 (4.6%, two from first MRE, and two from second MRE) corresponded to right and left central zones and two areas in the same patient (2.3%, one from first MRE, and one from second MRE) corresponded to the urethra. All areas of increased stiffness seen on both first MRE and second MRE were the same areas in both examinations.

**Table 5:** MRE findings of all patients in the study Suggest you uncouple first MRE from second in order to have complete widths for columns

No	Prostate volume	First MRE								Second MRE								Mean stiffness from first and second MRE
		No. of MRE slice	Mean stiffness (SD)	Min stiffness	Max stiffness	No. focal area of increased stiffness				No. of MRE slice	Mean stiffness (SD)	Min stiffness	Max stiffness	No. focal area of increased stiffness				
						Glandular	Stromal	Mixed	Other					Glandular	Stromal	Mixed	Other	
1	63.4	3	4.52 (2.09)	1.22	13.60	0	1	2	0	3	4.39 (1.63)	1.15	6.80	0	1	2	0	4.46
2	53.1	3	4.39 (1.35)	1.62	8.40	0	2	0	3 (2 central zones, 1 urethra)	3	4.28 (1.23)	1.72	8.54	0	2	0	3 (2 central zones, 1 urethra)	4.34
3	68.1	4	3.23 (0.93)	1.51	6.5	0	2	0	0	4	3.47 (0.91)	1.57	6.05	0	2	0	0	3.35
4	58.7	4	4.77 (1.78)	1.76	9.15	0	3	0	0	3	4.24 (1.41)	1.63	8.56	0	3	0	0	4.51
5	239.7	5	6.16 (2.03)	1.53	12.34	0	6	2	0	5	5.75 (1.76)	1.75	11.05	0	6	2	0	5.96
6	72.5	3	3.95 (1.47)	1.41	12.47	1	1	3	0	3	3.56 (1.23)	1.45	8.60	1	1	3	0	3.76
7	161.2	4	4.94 (1.94)	1.73	12.81	0	1	6	0	4	4.76 (1.74)	1.66	10.70	0	1	6	0	4.85
8	51.3	3	4.00 (2.21)	1.44	19.33	1	1	1	0	3	3.97 (2.05)	1.30	23.26	1	1	1	0	3.99
9	51.2	2	4.69 (1.91)	1.42	10.93	0	0	1	0	2	3.53 (0.81)	2.32	5.64	0	0	2	0	4.11
10	81.5	4	4.70 (2.32)	1.19	14.97	0	5	1	0	4	4.60 (2.49)	1.05	15.55	0	5	1	0	4.65

For analysis of test–retest reliability of tissue stiffness using ICC, it was found that mean stiffness had good overall reproducibility (ICC 0.82). Maximum stiffness (ICC 0.74) and SD of the stiffness (0.65) had moderate overall reproducibility. Minimum stiffness had poor reproducibility (ICC 0.42). For analysis of the reliability of number of acquired MRE slices and number of areas of increased stiffness on stiffness images, it was found that there was excellent reliability between the two examinations (ICC 0.93 and 0.99).

For per-patient results (Tables 4, 5), the patient No#5 was the only one who had BPH type 5. His MRI showed equal glandular-stromal components. He had the largest size of prostate gland (239.7 ml), the highest number of acquired MRE slices (five slices), and the highest average tissue stiffness (5.96 kPa). His PSA level was 11.0 ng/ml (PSAD 0.04 ng/ml^2^).

Patient No#10 had the highest PSA and PSAD levels, 26.6 ng/ml and 0.33 ng/ml^2^. He had the third-largest size of prostate gland (81.5 ml) and the third-highest average tissue stiffness (4.65 kPa). He had a BPH with type 3 and a predominant stromal component.

Patient No#3 had the lowest PSA (2.11 ng/ml) and PSAD (0.03 ng/ml^2^) levels. He had the lowest tissue stiffness too.

Patient No#9 had the lowest number of acquired slices and the smallest size of prostate gland, 51.2 ml.

## Discussion

This study showed that the MRE of the prostate gland using an external air driver with a frequency of 60 Hz is successful in evaluating tissue stiffness. All ten patients could well tolerate the mechanical vibration of the driver without any complications. This implies that the MRE of the prostate gland is a safe noninvasive method that could be added to conventional MRI. It was also possible to obtain the wave images which passed through the prostate gland and measured the tissue stiffness from the MRE examinations of all ten patients.

The mean tissue stiffness of the whole prostate gland in this study was 4.40 ± 0.71 kPa when compared with the previous MRE studies of J. Kemper et al. [12] He performed MRE using an external driver attached to the pubic bone by a 1.5 T scanner in seven healthy volunteers at a vibration frequency of 85 Hz and reported the mean values of elasticity inside the peripheral zone and central zone that were 3.3 ± 0.5 kPa and 2.2 ± 0.3 kPa, which were lower than the current study stiffness mean value. After that Sahebjavaher et al. studied MRE using a 3.0 T scanner transperineal electromechanical transducer at a frequency of 70 Hz in six healthy volunteers and found mean shear stiffnesses of 11.5 ± 2.9, 13.8 ± 4.5 and 13.2 ± 5.0 kPa for the peripheral, central and transition zones [13]. That study showed a much higher value of stiffness than in this present study, although the study was performed in healthy volunteers. This might be caused by the different electromechanical transducer in this study and an air driver as in the current study, a different wave frequency and different setting of volunteers. The recent study of prostate MRE was performed by Dittmann F et al. to assess the elasticity of prostate gland in 12 healthy volunteers using a 1.5 T scanner with MRE and three externally placed pressurized-air drivers at vibration frequencies of 60, 70, and 80 Hz. They found the shear wave speed of the entire prostate gland at a frequency of 60 Hz was 2.21 ± 0.22 m/s and there was no significant differences of values of shear wave speed between the peripheral zone (2.23 ± 0.20 m/s) and the central gland (2.18 ± 0.26 m/s) [14]. The shear wave speed (*V*_*s*_) to shear modulus (*µ, reported in kPa*) conversion using the equation *µ* = *ρV*_*s*_^2^ (*ρ* = soft tissue density, assumed to be 1,000 kg/m^3^) was used. So the mean stiffness values of the entire prostate gland, peripheral zone, and central gland from Dittmann’s study were 5.02, 4.97, and 4.75 kPa, which were slightly higher than in the present study. There were limitations to compare the results of the current study to other previous studies due to differences in MR scanner, MRE hardware, protocol, technique, population, and post-processing software. From the present results, it can be recommended that further studies to evaluate the reliability of MRE in different settings should be done.

For evaluation of test–retest reliability without repositioning of patients and driver, this study achieved good reproducibility (ICC 0.82) for mean stiffness and moderate reproducibility for maximum and SD of stiffness (ICC 0.74 and 0.65). Minimum stiffness had poor reproducibility (ICC 0.42). The different values between two tests might be caused by inhomogeneous waves traversing the prostate gland which was is a small organ located deeply in the pelvis and the movement of adjacent organs such as bowel peristalsis which might affect imaging acquisition. Excellent reproducibility was found for several acquired MRE slices (ICC 0.93) which mainly depended on the size of prostate gland, especially the prostate height. For analysis of the number of areas of increased stiffness on stiffness images, this study also found excellent reliability between two examinations (ICC 0.99). The study, however, did not reposition the subjects and actuators which might induce small changes in wave patterns and wave amplitudes. As the result of reproducibility analysis in the study of Dittmann F et al., they found good test–retest reproducibility despite repositioning of subjects and actuators between measurements (ICC = 0.88 and 0.78 in the central gland and peripheral zone) [14].

For the analysis of MRI findings, there was a nearly equal proportion of BPH components in this study; four patients with predominated glandular component, three patients with predominated stromal component, and three patients with equal glandular and stromal components. Most patients (90%) had a bilateral TZ and retrourethral enlargement pattern (type 3) and only one patient (10%) had a pedunculated with bilateral TZ and retrourethral enlargement pattern (type 5). These results were potentially the same as the Randall et al. classification [11]. There were no patients with another pattern of BPH in this study which might be caused by too small a population in the study.

For the analysis of focal areas of increased stiffness seen on stiffness images, about half of them (50.6%) corresponded to a stromal component on T2W images and more than one-third (37.9%) correspond to mixed glandular and stromal components. Additionally, it was found that the glandular predominant group tended to have a lower maximal stiffness than when stromal predominant or equal in its glandular-stromal component. These results supported the review of Wasserman et al. [4] that the stromal component in BPH leads to an increased resistance of prostatic parenchyma causing an increase in tissue stiffness.

Most other findings found in MRI were minimal ascites in the pelvic cavity, a small area of hemorrhage in the prostate gland and small prostatic utricle cysts which were not corresponding to areas of increased stiffness on stiffness images. Therefore, it can be assumed that these findings might not cause an increase in tissue stiffness. The study found, however, that some features which were seen on patients with high mean stiffness values including prostatic calcification, type-5 BPH pattern and the large prostate volume, it was possible to make an hypothesis that these features might be associated with an increase in tissue stiffness. Further study with more subjects would give more information about this hypothesis.

To the best of our knowledge, this study is the first study to exclusively describe MRE of the prostate gland in Thailand. There are several limitations to the study. Firstly, the study was a prospective study which had too small a number of subjects. Patients recruited into the study were less than was expected because of the Covid-19 situation in the country. Secondly, there might be a selection biases especially in the population-based selection from BPH patients with high PSA levels who were requested to have an MRI to screen for prostate cancer. Other patients with BPH may have more findings. Thirdly, the study had no healthy volunteers to compare the results with which would make a more reliable study. Fourthly, it did not demonstrate the pathological diagnosis which is the gold standard for diagnosis of BPH and its correlation to MRI and MRE findings. Lastly, it tried to perform prostate MRE using the drivers and post-processing software which were developed for measuring stiffness in the liver for the staging of liver fibrosis. The protocol might not be suitable for measuring stiffness in prostate gland so the results in the study might not represent valid tissue stiffness values. It is believed, however, that the results of the study would be beneficial for the development of an MRE protocol as an additional diagnostic tool for evaluating prostate disease and a guide to study more about prostate MRE in the future.

## Conclusions

Prostate MRE is a useful noninvasive reproducible diagnostic tool for evaluating prostate tissue stiffness in both qualitative and quantitative assessments. The mean prostate tissue stiffness from MRE in patients with benign prostatic enlargement in this study was 4.40 ± 0.71 kPa. Some MRI features might be associated with increased tissue stiffness.

## Data Availability

All data and material in this study are available to your request.

## Abbreviations

BPH: Benign prostatic hyperplasia
FFE: Fast field echo
ICC: Interclass correlation coefficient
MRE: Magnetic resonance elastography
MRI: Magnetic resonance imaging
PI-RADS: Prostate imaging reporting and data system
PSA: Prostatic specific antigen
TZ: Transition zone
